# Supplementary material for: Atmospheric deposition and river runoff stimulate the utilization of dissolved organic phosphorus in coastal seas
Source: Nat Commun. 2024 Jan 30;15:658. doi: 10.1038/s41467-024-44838-7 (PMC10828365; doi:10.1038/s41467-024-44838-7)
Supplement: Supplementary file 1 — Supplementary Information [file 41467_2024_44838_MOESM1_ESM.pdf]

Atmospheric deposition and river runoff stimulate the utilization of dissolved organic phosphorus in coastal seas

Haoyu Jin[1,2,3], Chao Zhang[1,2*], Siyu Meng[1,2,3], Qin Wang[1,2], Xiaokun Ding[4], Ling Meng[5,6], Yunyun Zhuang[1,2], Xiaohong Yao[1,2], Yang Gao[1,2], Feng Shi[1,2], Thomas Mock[3*] and Huiwang Gao[1,2*]

[1] Frontiers Science Center for Deep Ocean Multispheres and Earth System, and Key Laboratory of Marine Environment and Ecology, Ministry of Education of China, Ocean University of China, Qingdao, 266100, China.

[2] Marine Ecology and Environmental Science Laboratory, Laoshan Laboratory, Qingdao, 266071, China.

[3] School of Environmental Sciences, University of East Anglia, Norwich Research Park, Norwich, NR4 7TJ, United Kingdom.

[4] School of Ocean, Yantai University, Yantai, 264005, China.

[5] CAS Key Laboratory of Coastal Environmental Processes and Ecological Remediation, Yantai Institute of Coastal Zone Research, Chinese Academy of Sciences, Yantai 264003, China

[6] University of Chinese Academy of Sciences, Beijing 100049, China

Supplementary information

Supplementary methods

Particulate organic carbon (POC) and particulate organic nitrogen (PON) analysis

Mostly at the beginning, middle and the end of the incubations, 300 - 600 mL water samples (mixed equivalent 3 replicates) were collected on pre-combusted GF/F filters (Whatman) and stored at -20 °C. The filters and 4 blanks were then acidified with hydrochloric acid, and then dried in 60 °C for 24 hours. They were placed into tin capsules and stored in a desiccator until analysis. The tin capsules were loaded into the elemental analyzer and measuring POC and PON[1]. To ensure accuracy, the instrument is calibrated with acetanilide standards before analysis. The detection limit of the POC and PON are 0.02 and 0.04 $\mu g \cdot L^{-1}$, respectively.

Additional microcosm experiments

During summer 2023, we conducted an additional laboratory microcosm experiment using seawater collected in the Yellow Sea (Y4 Summer, 37.00 °N, 123.83 °E) to provide independent evidence of the Anthropogenic Nitrogen Pump. Surface seawater was collected using Teflon-coated Niskin bottles assembly on a SeaBird CTD and pre-filtered through 200 μm acid-washed nylon mesh to eliminate large zooplankton. Seawater was then put into a clean bucket, stored in a dark place at room temperature, and brought back to the laboratory within 24 hours. Seawater samples were then transferred into transparent polycarbonate bottles (2 L bottle experiments, Nalgene 2015-2000), and instantly spiked with nutrients and trace metal (Fe: $FeCl_3$, 2 nM; N: KNO_3, 2 μM; P: KH_2PO_4, 0.2 μM; N+P: 2+0.2 μM). Three parallel experiments (n = 3 samples) were set up for each treatment. The bottles were placed in a constant temperature incubator. The incubation lasted for 6 days at 22 °C and illuminated on 12 (Light):12 (Dark) cycle by 4500 lux. Besides, two on-board microcosm experiments

conducted in the East China Sea (E2 Autumn, 31.86 °N, 124.56 °E) and Yellow Sea (Y5 Autumn, 37.90 °N, 123.15 °E) during the autumntime of 2020 were also used for supporting our results. APA was measured in these three experiments using the same methods as used for the earlier onboard experiments.

Supplementary tables & figures

Supplementary Table 1 | Sampling condition and chemical composition of aerosols and riverine water

Samples (date)	Nutrients			Trace Metal			Meteorological Condition		
	DIN	DIP	DIN:DIP	Fe	Zn	Al	Relative humidity	Visibility	Air Quality Index
	μmol·m^{-3}			nmol·m^{-3}			%	km	(AQI)
Aerosol 1 (16/01/2018)	2.14	0.66×10^{-3}	3280	0.98	7.57	3.36	77	2.8	224
Aerosol 2 (14/01/2019)	2.62	3.43×10^{-3}	769	2.24	4.90	6.63	74	0.6	313
Aerosol 3 (13/07/2019)	0.99	8.36×10^{-3}	118	0.77	1.57	3.78	69	9.7	73
	μmol·L^{-1}								
Riverine water (08/03/2019)	330.54	0.65	509						

Supplementary Table 2 | Physical and chemical properties of the seawater used for the microcosm experiments. Water on station Y1 has been sampled in the surface and the deep chlorophyll *a* maximum layer (DCM). Spr = spring; Sum = summer; DIN = dissolved inorganic nitrogen; DIP = dissolved inorganic phosphorous; DOP = dissolved organic phosphorous; APA = activity of the alkaline phosphatase.

Stations	E1	$Y3_{Spr}$	Y1		Y2	$Y3_{Sum}$	$B1_{Spr}$	$B1_{Sum}$
Sampling date (d/m/y)	08/04/2019	29/03/2018	26/07/2019		02/08/2018	25/07/2018	02/05/2019	29/07/2019
Bottom depth (m)	41	70	65		74	70	27	27
Water Depth (m)	3~5	3~5	3~5	~18 (DCM)	3~5	3~5	3~5	3~5
Temperature (℃)	15	10	28	17	32	28	13	29
DIN (μM)	9.83	0.49	1.53	1.99	0.27	0.23	0.68	3.60
DIP (μM)	0.07	0.04	0.01	0.11	0.02	0.01	0.01	0.03
DOP (μM)	0.10	0.15	0.20	0.17	0.21	0.25	0.13	0.20
$Si(OH)_4$ (μM)	3.18	0.79	1.69	3.10	1.02	1.04	0.20	8.47
DIN:DIP	151	12	200	18	15	18	127	107
Chl *a* ($\mu g \cdot L^{-1}$)	0.43	4.47	0.47	1.21	0.14	1.16	3.07	2.94
APA ($nM\ P \cdot h^{-1}$)	0.4	/	26.2	2.1	9.0	15.5	0.9	179.1

Supplementary Table 3 | Recent studies on activity of alkaline phosphatase (APA) and its correlation to dissolved inorganic phosphorous (DIP) concentration in different regions. The DIP threshold is the critical DIP concentration below which a high APA takes place.

Reference	Study site	Location	APA (nM P·h^{-1})	DIP threshold (μM)
This study	East China Sea and Yellow Sea	Coastal sea and mid-shelf	0.3-126.4	0.02
	Bohai Sea	Coastal seas	0.9-15.0<br>129.4-564.7	Not found (spring)<br>0.024 (summer)
(Zhang et al. 2018)[2]	Daya bay	Coastal seas	47.2-1692.2	0.2
(Dyhrman and Ruttenberg 2006)[3]	Off northern Oregon	Coastal seas	0.5-3.2	0.5
(Mahaffey et al. 2014)[4]	Eastern subtropical Atlantic	Open ocean	0-13.7	0.03
(Lomas et al. 2010)[5]	Sargasso Sea	Open ocean	0-14.0	0.01
(Suzumura et al. 2012)[6]	Subtropical Pacific	Open ocean	0.2-0.8	0.02

Supplementary Table 4 | Microcosm experimental design

Stations	Treatment	Concentration enhanced after treatment				
		Nutrients		Trace Metal		
		DIN	DIP	Fe	Zn	Al
		μmol·L^{-1}		nmol·L^{-1}		
All stations*	DIN	2				
	DIP		0.2			
	DIN+DIP	2	0.2			
	DIN+Fe	2		2		
	DIN+DIP+Fe	2	0.2	2		
$Y3_{Spr}$, $Y2_{Sum}$, $Y3_{Sum}$	Aerosol 1-low	0.5	0.16×10^{-3}	0.23	1.77	0.79
	Aerosol 1-medium	1	0.31×10^{-3}	0.46	3.54	1.57
	Aerosol 1-high	2	0.62×10^{-3}	0.92	7.07	3.14
E1, $B1_{Spr}$	Aerosol 2-low	0.5	0.66×10^{-3}	0.43	0.94	1.27
	Aerosol 2-medium (E1 only)	1	1.31×10^{-3}	0.86	1.88	2.53
	Aerosol 2-high	2	2.62×10^{-3}	1.71	3.75	5.06
Y1, Y1-DCM, $B1_{Sum}$	Aerosol 3-low	0.5	4.22×10^{-3}	0.37	0.76	1.82
	Aerosol 3-high	2	16.89×10^{-3}	1.47	3.03	7.27
$B1_{Spr}$, $B1_{Sum}$*	River-low	0.5	0.98×10^{-3}			
	River-high	2	3.93×10^{-3}			

*** DIN+DIP+Fe addition was only conducted at $Y3_{Spr}$, Y2, $Y3_{Sum}$ in 2018, DIN+Fe was only conducted at E1, $B1_{Spr}$, and $B1_{Sum}$ in 2019. DIP addition was not conducted at Y1 and Y1-DCM. River-low group was not conducted at $B1_{Spr}$.**

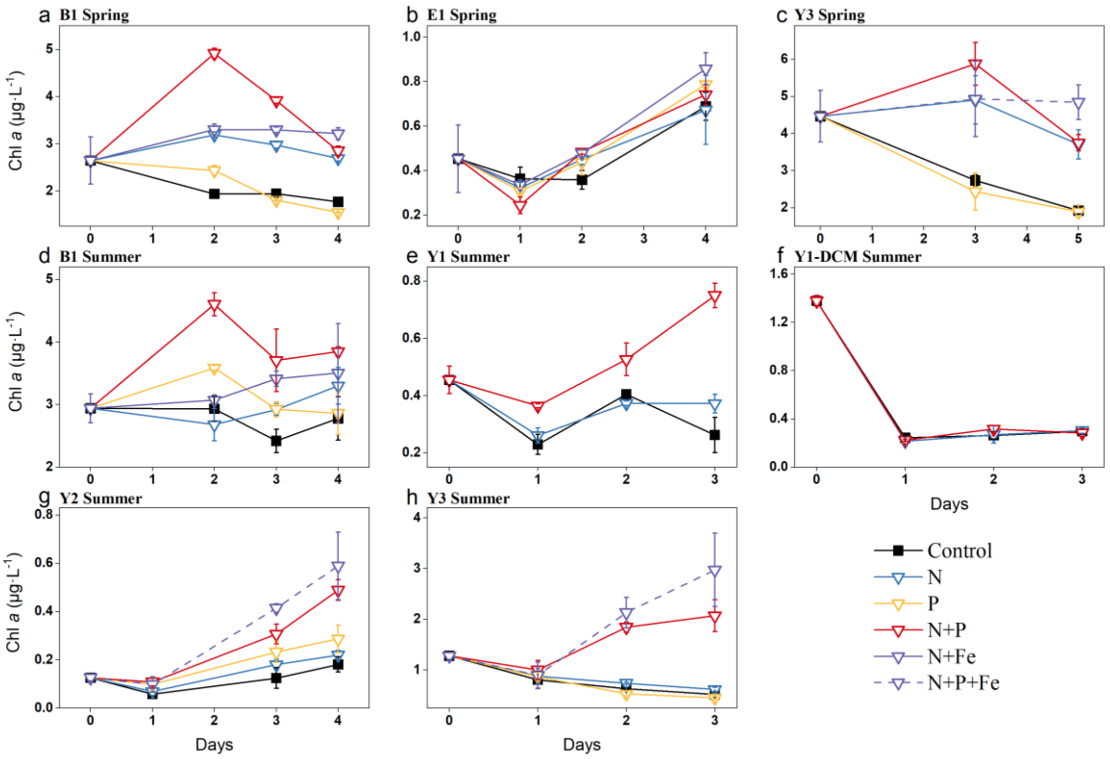

Supplementary Fig. 1 | Chlorophyll *a* (Chl *a*) concentrations during the incubations according to nutrient additions. Chl *a* concentrations in spring at stations B1 (a), E1 (b) and Y3 (c). Chl *a* concentrations in summer at stations B1 (d), Y1 (e), Y1-DCM (f), Y2 (g) and Y3 (h). N = nitrate, P = phosphate, Fe = iron. Data are presented as mean values +/- SD, *n* = 3 samples. Source data are provided as a Source Data file.

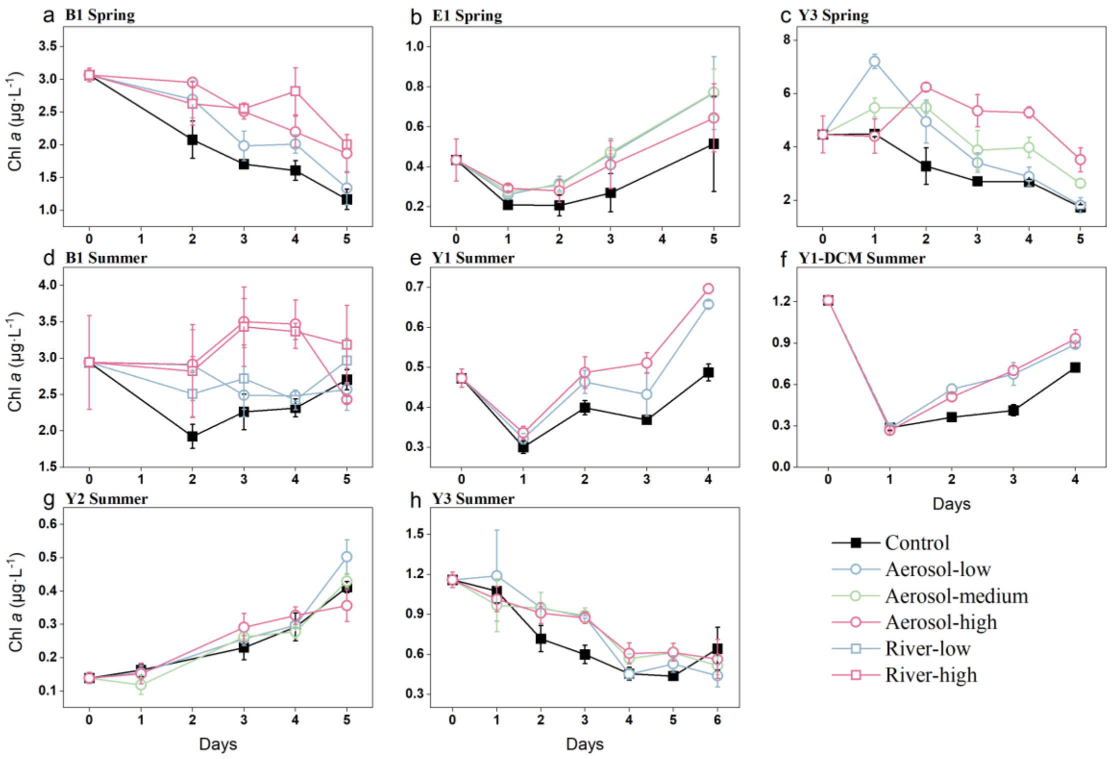

Supplementary Fig. 2 | Chlorophyll *a* (Chl *a*) concentrations during the incubations according to aerosol and riverine water additions. Chl *a* concentrations in spring at stations B1 (a), E1 (b) and Y3 (c). Chl *a* concentrations in summer at stations B1 (d), Y1 (e), Y1-DCM (f), Y2 (g) and Y3 (h). Data are presented as mean values +/- SD, *n* = 3 samples. Source data are provided as a Source Data file.

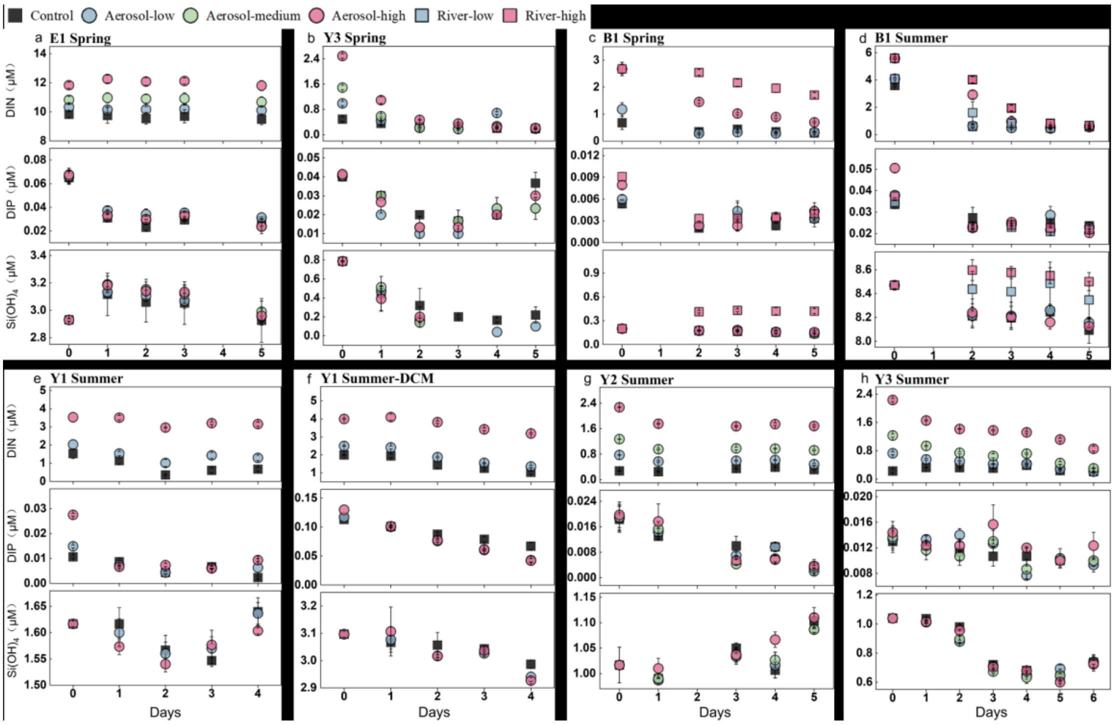

Supplementary Fig. 3 | Inorganic nutrient concentrations during the incubations. Nutrient concentrations in spring at stations E1 (a), Y3 (b) and B1 (c). Nutrient concentrations in summer at stations B1 (d), Y1 (e), Y1-DCM (f), Y2 (g) and Y3 (h). DIN (NO_3^-+NO_2^-+NH_4^+) and DIP represent dissolved inorganic nitrogen and dissolved inorganic phosphorus, respectively. The $Si(OH)_4$ concentration of aerosol addition groups during the days 3 – 5 at Y3 Spring (except aeosol-low group in 4 – 5 days) were lower than its dectective limit (≤ 0.03 μM). Data are presented as mean values +/- SD (n = 3 samples). Source data are provided as a Source Data file.

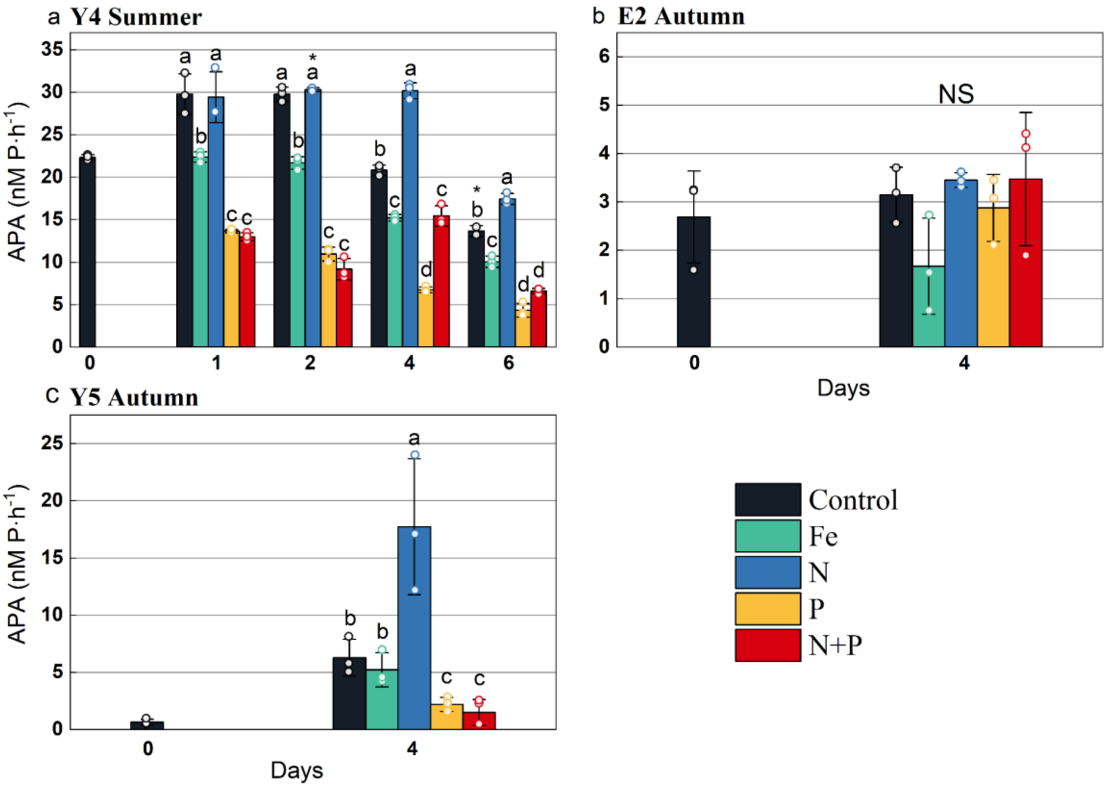

Supplementary Fig. 4 | Activity of alkaline phosphatase (APA) in response to iron and nutrient additions. a APA responses in the microcosm experiment conducted in laboratory using the surface seawater collect in the Yellow Sea during the summertime of 2023. **b and c** APA responses in the microcosm experiments conducted in the East China Sea (E2 Autumn) and the Yellow Sea (Y5 Autumn) during the autumntime of 2020. Significant differences ($p < 0.05$) are indicated by different letters and NS = not significant (one-way ANOVA and Turkey HSD post hoc test). Data are presented as mean values +/- SD, n = 3 samples for all except where indicated by an asterisk (n = 2 samples). Source data are provided as a Source Data file.

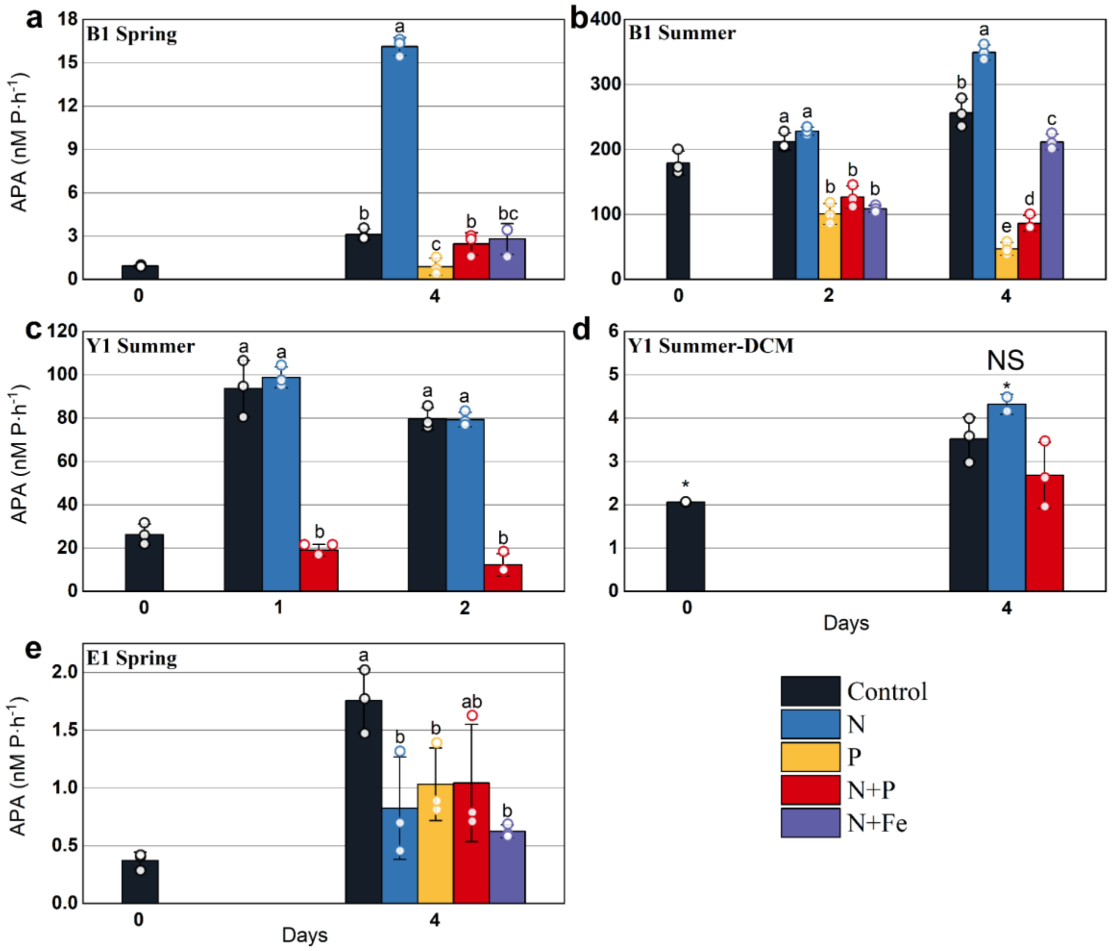

Supplementary Fig. 5 | Activity of alkaline phosphatase (APA) in response to nutrient amendments during the microcosm experiments in 2019. APAs in the Bohai Sea in spring (a) and summer (b). APAs in the Yellow Sea in summer (c,d) and the East China Sea in spring (e). APA of different groups were compared using one-way ANOVA and Turkey HSD post hoc tests. Significant differences are indicated by different letters ($p < 0.05$). NS = not significant. Data are presented as mean values +/- SD, $n = 3$ samples for all except where indicated by an asterisk ($n = 2$ samples). Source data are provided as a Source Data file.

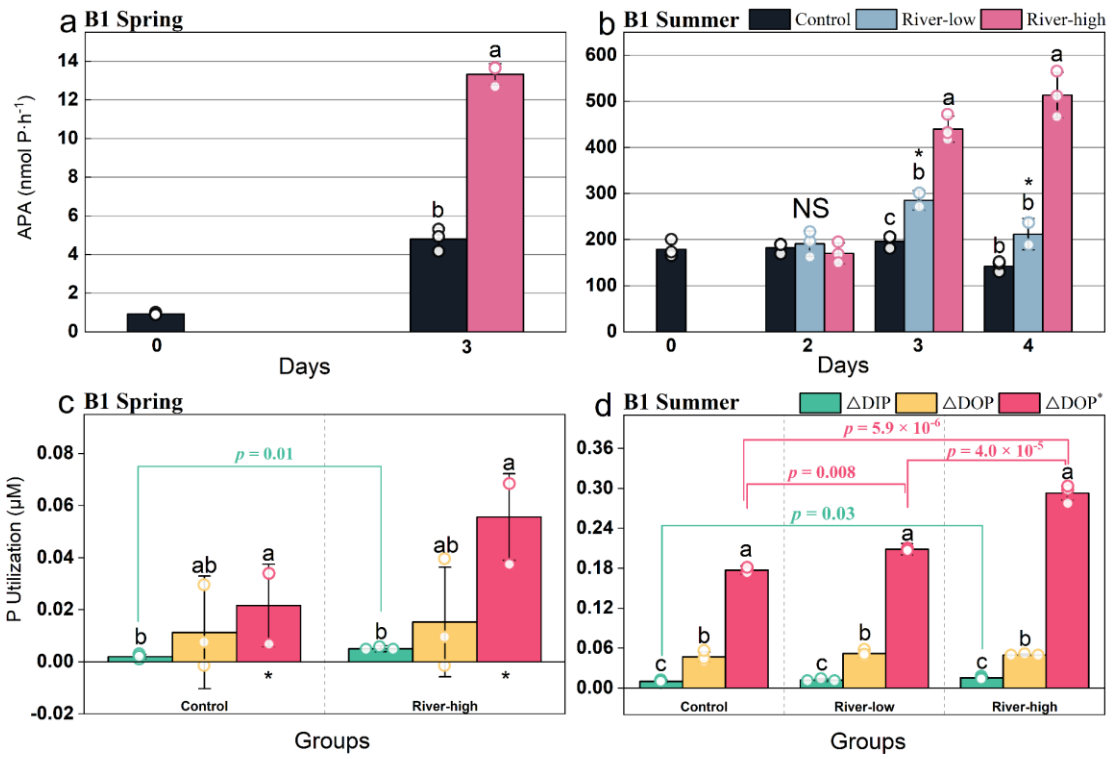

Supplementary Fig. 6 | Activity of alkaline phosphatase (APA) and the uptake of phosphorus (P) in response to riverine water additions. a and b APAs. For APAs in the same day of different groups, significant differences (p < 0.05) are indicated by different letters and NS = not significant (one-way ANOVA and Turkey HSD post hoc test). Data are presented as mean values +/- SD, n = 3 samples for all except where indicated by an asterisk (n = 2 samples). **c and d** The utilization of dissolved inorganic phosphorus (△DIP), the net utilization of dissolved organic phosphorus (△DOP), and the potential maximum DOP utilization (△DOP* = △DIN/16-△DIP). For DOP utilization, △DOP was calculated from the change of DOP concentration during the incubations, △DOP* was calculated by the DIN (△DIN) and DIP (△DIP) utilization during the incubations, and 16:1 is the Redfield ratio of N:P. Significant differences (p < 0.05) between different P utilization (△DIP, △DOP, △DOP*) in the same group are indicated by different letters, and significant differences (p < 0.05) for P utilization between treatment and control groups are indicated with connecting coloured lines and p values (one-way ANOVA and Turkey HSD post hoc test). Data are presented as mean values +/- SD, n = 3 samples for all except where indicated by an asterisk (n = 2 samples). Source data are provided as a Source Data file.

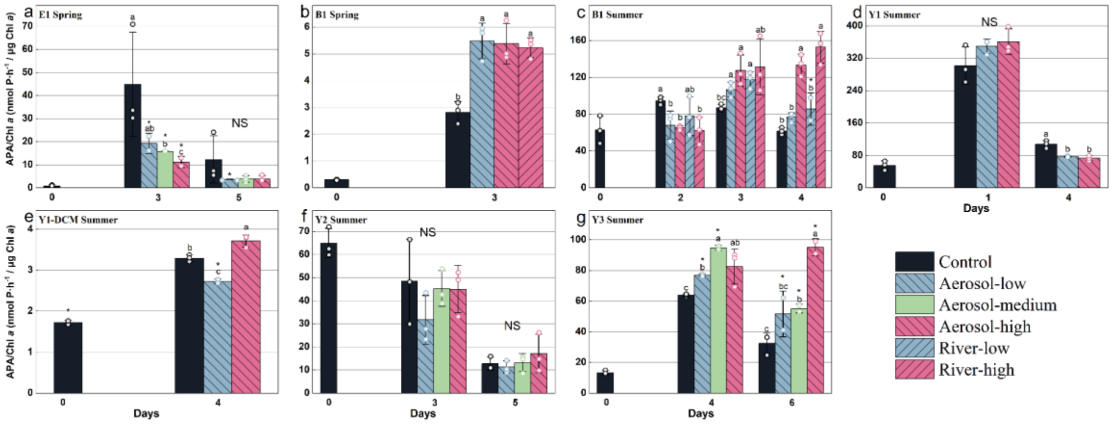

Supplementary Fig. 7 | The ratios of activity of alkaline phosphatase to chlorophyll *a* (APA / Chl *a*) in response to aerosol and riverine water additions. APA / Chl *a* ratios in the East China Sea in spring (a), in the Bohai Sea in spring (b) and in summer (c). APA / Chl *a* ratios in the Yellow Sea in summer (d,e,f,g). Significant differences ($p < 0.05$) are indicated by different letters and NS = not significant (one-way ANOVA and Turkey HSD post hoc test). Data are presented as mean values +/- SD, $n = 3$ samples for all except where indicated by an asterisk ($n = 2$ samples). Source data are provided as a Source Data file.

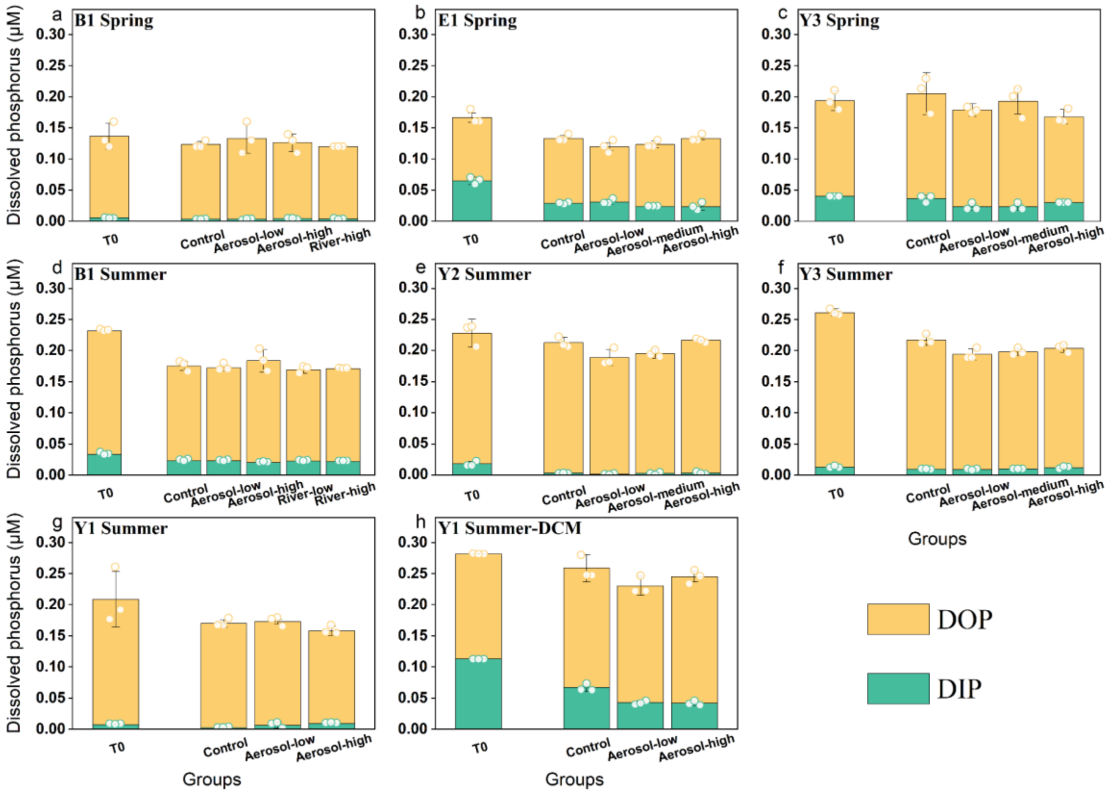

Supplementary Fig. 8 | Concentrations of total dissolved phosphorus (TDP), dissolved inorganic phosphorus (DIP), and dissolved organic phosphorus (DOP) at the beginning (T0) and end of the incubations. Concentrations in spring at stations B1 (a), E1 (b) and Y3 (c). Concentrations in summer at stations B1 (d), Y2 (e), Y3 (f), Y1 (g) and Y1-DCM (h). TDP = DIP + DOP. Data are presented as mean values +/- SD, *n* = 3 samples. Source data are provided as a Source Data file.

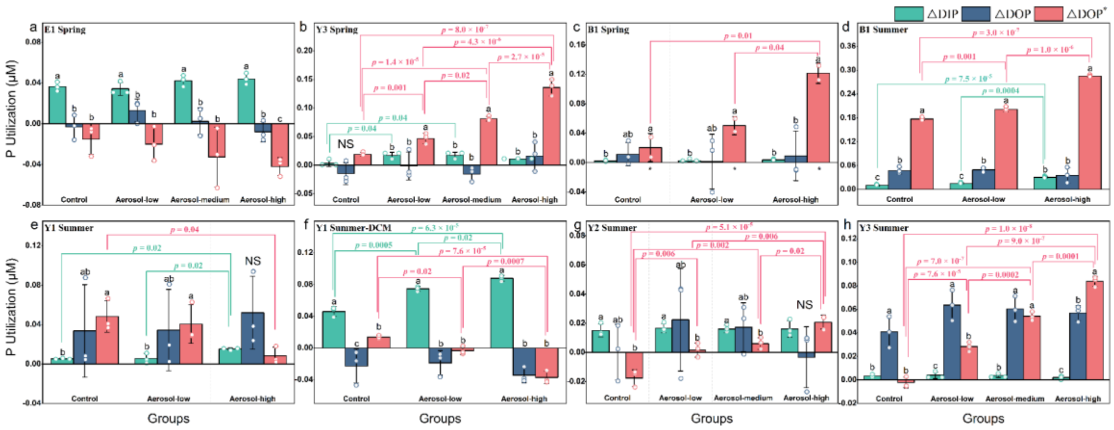

Supplementary Fig. 9 | The uptake of phosphorus (P) in response to aerosol additions. The utilization of dissolved inorganic phosphorus (△DIP), the net utilization of dissolved organic phosphorus (△DOP), and the potential maximum DOP utilization (△DOP*) in the microcosm experiments in spring (a, b, c) and in summer (d, e, f, g, h). For DOP utilization, △DOP was calculated from the change of DOP concentration during the incubations, △DOP* (△DIN/16-△DIP) was calculated by the DIN (△DIN) and DIP (△DIP) utilization during the incubations, and 16:1 is the Redfield ratio of N:P. Significant differences ($p < 0.05$) between different P utilization (△DIP, △DOP, △DOP*) in the same group are indicated by different letters, and significant differences ($p < 0.05$) for P utilization between treatment and control groups are indicated with connecting coloured lines and p values (one-way ANOVA and Turkey HSD post hoc test). Data are presented as mean values +/- SD, $n = 3$ samples for all except where indicated by an asterisk ($n = 2$ samples). Source data are provided as a Source Data file.

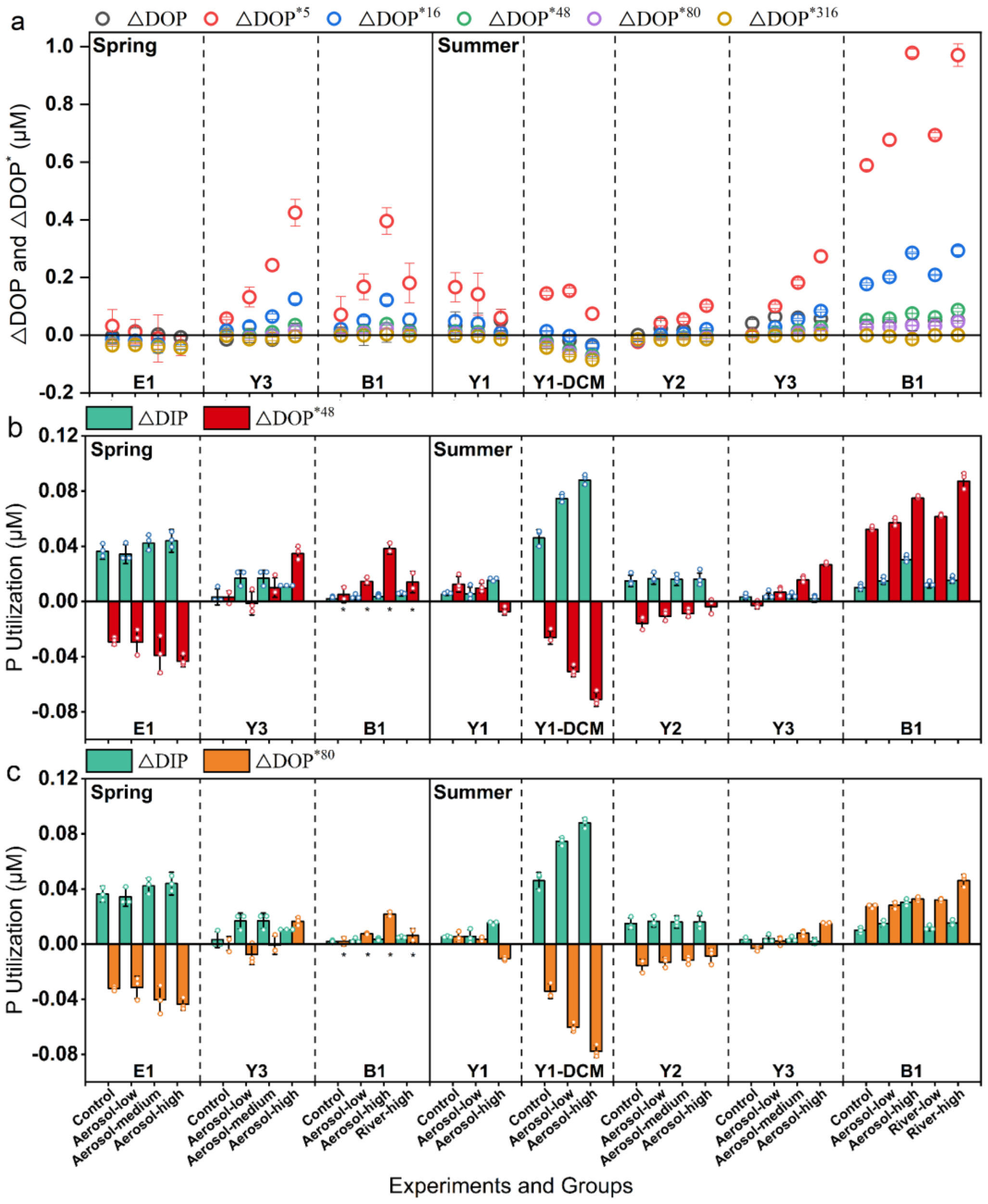

Supplementary Fig. 10 | DOP utilization. a Estimated utilization of dissolved organic phosphorus (DOP) using different N:P uptake ratios. △DOP represents the change of DOP concentrations during the incubations. △DOP^{*x} represents potential total DOP utilization (△DIN/x-△DIP) using lowest particulate N:P ratio in the nature seawater (5), Redfield ratio of N:P (16), 3-folds Redfield ratio of N:P (48), 5-folds Redfield ratio of N:P (80), and higest particulate N:P ratio in the nature seawater (316). △DIN and △DIP represent the utilization of dissolved inorganic nitrogen and dissolved inorganic phosphorus, respectively. **b and c** Comparison of DIP utilization and Estimated DOP utilization (△DOP^{*48}, △DOP^{*80}). △DIP represents the change of DIP concentrations during the incubations. Data are presented as mean values +/- SD, *n* = 3 samples for all except where indicated by an asterisk (*n* = 2 samples). Source Data are provided as a Source Data file.

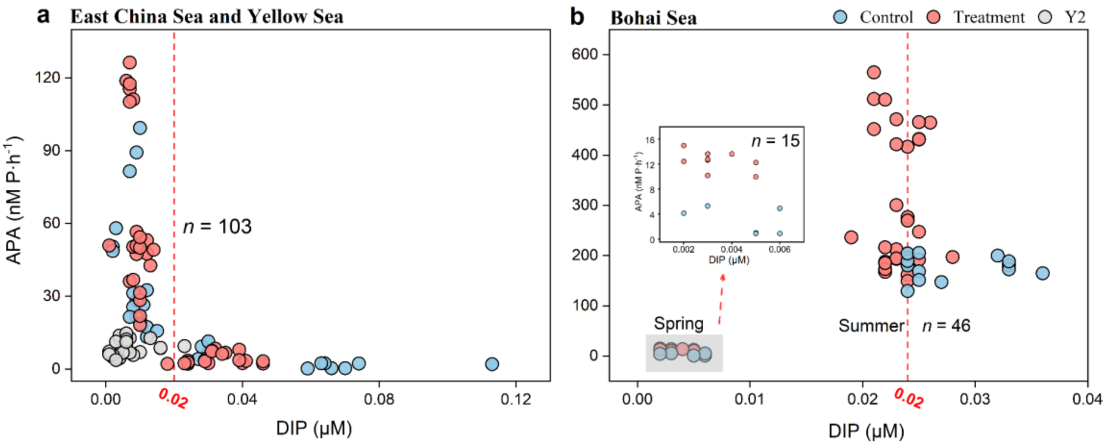

Supplementary Fig. 11 | Correlation between the activity of alkaline phosphatase (APA) and dissolved inorganic phosphorus (DIP) concentrations. a Correlation between APA and DIP in the East China Sea and Yellow Sea. **b** Correlation between APA and DIP in the Bohai Sea. Blue dots indicate data in the control groups, and red dots indicate data in the aerosol and riverine water treatment groups. The dashed lines indicate the DIP thresholds. DIP thresholds were calculated by segmented regression (R package, "segmented"). Grey dots in **a** indicate the data in the Y2 which was not included in segmented regression. Data covered by grey shade in **b** indicate data in the $B1_{Spr}$ which was not included in segmented regression. Insert in **b** shows data covered by grey shade. *n*, number of samples. Source data are provided as a Source Data file.

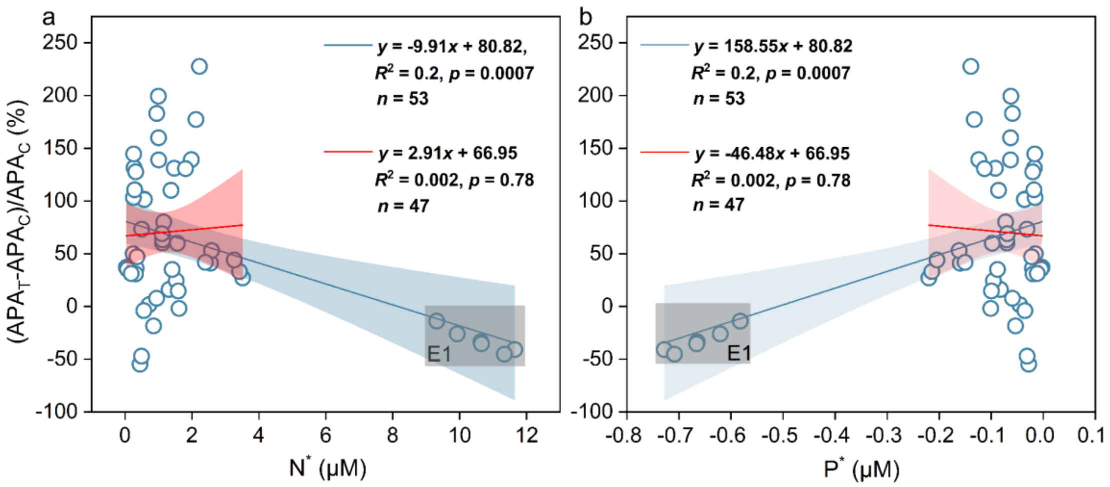

Supplementary Fig. 12 | Dissolved organic phosphorus (DOP) utilization indicated by the activity of alkaline phosphatase (APA) and its correlation with N* and P*. Correlation between the relative change of APA after treatment and the parameters N* (DIN 16 × DIP) (a) and P* (DIP DIN / 16) (b) , of which APA_C and APA_T represent APA on the day they began to enhance (mainly in the middle day of incubations) in the control and aerosol/riverine water treatments, respectively. DIN and DIP represent the concentration of dissolved inorganic nitrogen (N) and dissolved inorganic phosphorus (P), respectively. 16 represents the Redfield ratio of N:P. Positive N* indicates DIP deficiency, and negative P* indicates DOP consumption. The blue and red lines (best-fit line) with shades (95% confidence level) represent the results of the linear regression with and without data of E1 (covered by grey shades), respectively. *p* values from a two-sided test are shown. *n*, number of samples. Source data are provided as a Source Data file.

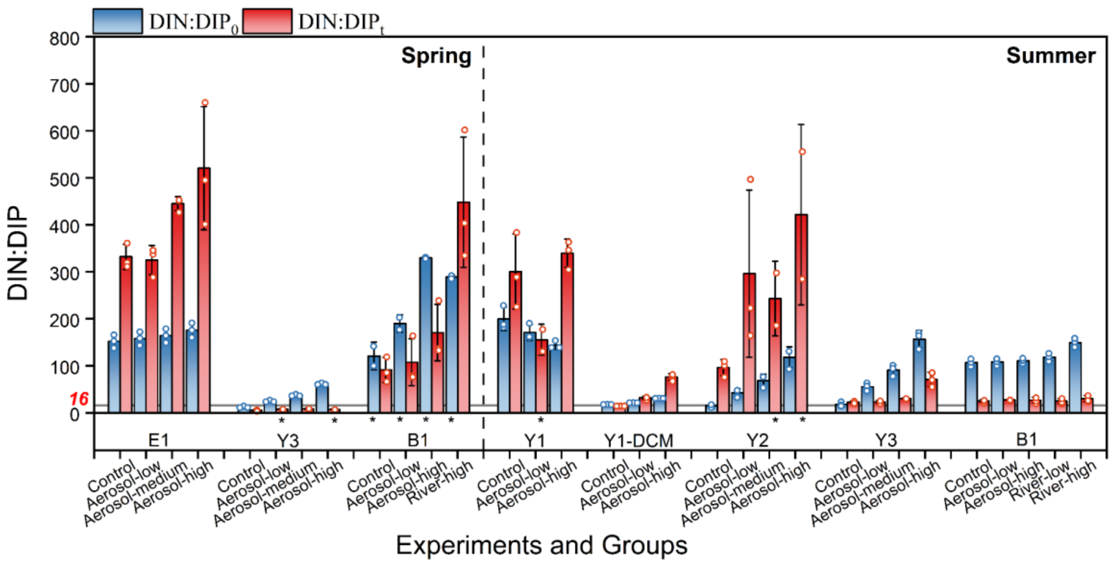

Supplementary Fig. 13 | The ratios of dissolved inorganic nitrogen to dissolved inorganic phosphorus (DIN:DIP) at the beginning (DIN:DIP$_0$) and end (DIN:DIP$_t$) of the incubations. The horizontal grey line at the bottom of the graph represents the N:P uptake ratio by phytoplankton (Redfield ratio: 16:1). Data are presented as mean values +/- SD, *n* = 3 samples for all except where indicated by an asterisk (*n* = 2 samples) (Fig. S3). Source data are provided as a Source Data file.

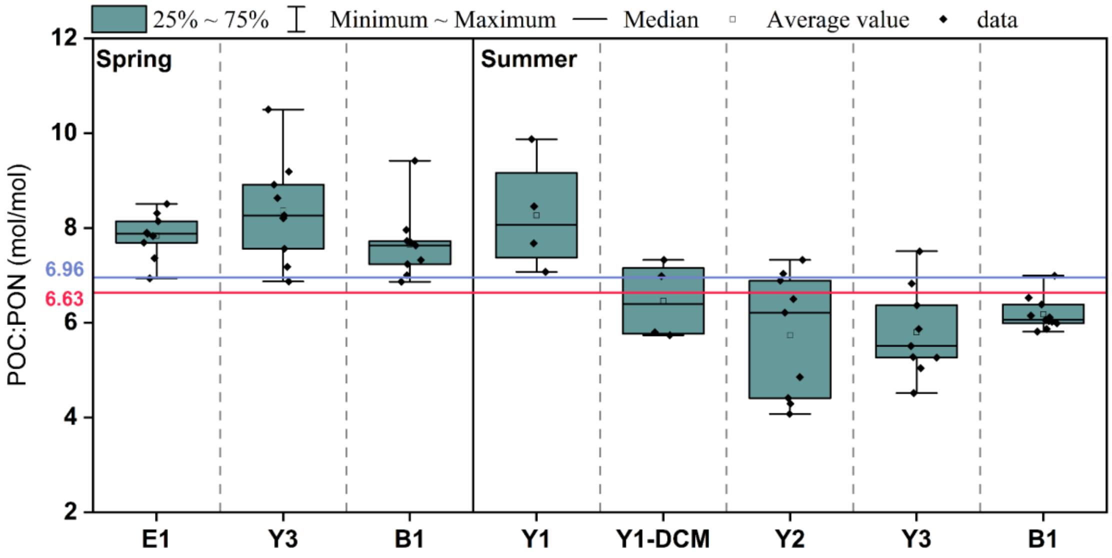

Supplementary Fig. 14 | Ratios of particulate organic carbon to particulate organic nitrogen (POC:PON) during the incubations. The lines across the boxes represent 25th (bottom), 50th (middle), and 75th (top) percentiles. The whisker caps represent minimum (bottom) and maximum (top) values. Blue and red lines represent the average POC:PON ratio in this study and Redfield ratio of carbon to nitrogen, respectively. Source data are provided as a Source Data file.

Supplementray References

1. Nieuwenhuize, J., Maas, Y. E. M. & Middelburg, J. J. Rapid analysis of organic carbon and nitrogen in particulate materials. *Mar. Chem.* **45**, 217-224 (1994).
2. Zhang, X., Zhang, J., Shen, Y., Zhou, C. & Huang, X. Dynamics of alkaline phosphatase activity in relation to phytoplankton and bacteria in a coastal embayment Daya Bay, South China. *Mar. Pollut. Bull.* **131**, 736-744 (2018).
3. Dyhrman, S. T. & Ruttenberg, K. C. Presence and regulation of alkaline phosphatase activity in eukaryotic phytoplankton from the coastal ocean: Implications for dissolved organic phosphorus remineralization. *Limnol. Oceanogr.* **51**, 1381-1390 (2006).
4. Mahaffey, C., Reynolds, S., Davis, C. E. & Lohan, M. C. Alkaline phosphatase activity in the subtropical ocean: insights from nutrient, dust and trace metal addition experiments. *Front. Mar. Sci.* **1**, (2014).
5. Lomas, M. W. et al. Sargasso Sea phosphorus biogeochemistry: an important role for dissolved organic phosphorus (DOP). *Biogeosciences* **7**, 695-710 (2010).
6. Suzumura, M., Hashihama, F., Yamada, N. & Kinouchi, S. Dissolved Phosphorus Pools and Alkaline Phosphatase Activity in the Euphotic Zone of the Western North Pacific Ocean. *Front. Microbiol.* **3**, (2012).
